# Supplementary material for: Immunotranscriptomic profiling the acute and clearance phases of a human challenge dengue virus serotype 2 infection model
Source: Nat Commun. 2021 May 24;12:3054. doi: 10.1038/s41467-021-22930-6 (PMC8144425; doi:10.1038/s41467-021-22930-6)
Supplement: Supplementary file 1 — Supplementary Information [file 41467_2021_22930_MOESM1_ESM.pdf]

Supplementary Table 1. Subset demographics.
Supplementary Table 2. List of reagents used.
Supplementary Figure 1 | DENV2 viremia after rDEN2Δ30 infection.
Supplementary Figure 2 | DENV2 serum neutralizing antibodies after rDEN2Δ30 infection.
Supplementary Figure 3 | Quantity and quality of cellular mRNA during rDEN2Δ30 infection.
Supplementary Figure 4 | Flow cytometric gating strategy
Supplementary Figure 5 | Titration of flow cytometry antibodies

Supplementary Table 1. Subset demographics.

	Parent study (n = 20)	*Gene expression subset (n = 11)*
Female	6 (30%)	2 (18%)
Male	14 (70%)	9 (82%)
Black	9 (45%)	5 (45%)
White	11 (55%)	6 (55%)
Baltimore	11 (55%)	6 (55%)
Vermont	9 (45%)	5 (45%)

Supplementary Table 2. List of reagents used.

REAGENT or RESOURCE		SOURCE	IDENTIFIER
Antibodies			
Anti-human CD3 (UCHT1) FITC-conjugated	0.25 µL/test	Biolegend	Cat. #300406 Lot #B279208
Anti-human CD4 (OKT4) BV510-conjugated	1 µL/test	Biolegend	Cat. #317444 Lot #B248141
Anti-human CD8 (RPA-T8) BV650-conjugated	0.5 µL/test	Biolegend	Cat. #301041 Lot #B275821
Anti-human CD14 (M5E2) BV711-conjugated	1 µL/test	Biolegend	Cat. #301837 Lot #B275829

Anti-human CD16 (3G8)<br>APC-Cy7-conjugated	0.25 µL/test	Biolegend	Cat. #302017<br>Lot #B295391
Anti-human CD19 (HIB19)<br>PE-Dazzle594-conjugated	0.5 µL/test	Biolegend	Cat. #302252<br>Lot #B277039
Anti-human CD25 (M-A251)<br>BV421-conjugated	0.5 µL/test	Biolegend	Cat. #356113<br>Lot #B301467
Anti-human CD27 (M-T271)<br>PE-Cy7-conjugated	0.125 µL/test	Biolegend	Cat. #356412<br>Lot #B279971
Anti-human CD38 (HIT2)<br>Alexa Fluor 647-conjugated	0.125 µL/test	Biolegend	Cat. #303514<br>Lot #B233813
Anti-human CD45RA (HI100)<br>BUV395-conjugated	0.25 µL/test	BD OptiBuild	Cat. #740298<br>Lot #0293615
Anti-human CD56 (NCAM16.2)<br>BUV563-conjugated	0.125 µL/test	BD Horizon	Cat. #612929<br>Lot #0044064
Anti-human CD57 (QA17A04)<br>BV605-conjugated	0.5 µL/test	Biolegend	Cat. #393303<br>Lot #270939
Anti-human CD127 (HIL-7R-M21)<br>BUV805-conjugated	0.25 µL/test	BD OptiBuild	Cat. #748486<br>Lot #0294340
Anti-human CD134 (ACT35)<br>BUV737-conjugated	0.125 µL/test	BD OptiBuild	Cat. #749286<br>Lot #0294339
Anti-human CD154 (24-31)<br>BV785-conjugated	0.5 µL/test	Biolegend	Cat. #310841<br>Lot #B264809
Anti-human HLA-DR (L243)<br>BV570-conjugated	2.5 µL/test	Biolegend	Cat. #307637<br>Lot #B314475
Anti-human IgM (MHM-88)<br>PerCP-Cy5.5-conjugated	0.5 µL/test	Biolegend	Cat. #314512<br>Lot #B231968
Anti-human CCR7 (2-L1-A)<br>APC-R700-conjugated	0.5 µL/test	BD Horizon	Cat. #566767<br>Lot #0283646
Anti-human CD279 (EH12.2H7)<br>PE-conjugated	0.5 µL/test	Biolegend	Cat. #329905<br>Lot #B252642
Human Trustain FcX blocker	5 µL/test	Biolegend	Cat. #422302<br>Lot #B313422
True-Stain Monocyte blocker	5 µL/test	Biolegend	Cat. #426102<br>Lot #B311012
Live/Dead Blue Viability Dye	0.5 µL/test	Invitrogen	Cat. #L23105 A<br>Lot #2214471
Brilliant stain buffer	50 µL/test	BD Horizon	Cat. #566349<br>Lot #9192749
Bacterial and Virus Strains			
Dengue virus serotype 1 (West Pacific 74)<br>WHO reference strain		Stephen Whitehead, National Institute of Allergy and Infectious Disease (NIAID)	Genbank AY145121
Dengue virus serotype 2 (New Guinea C)		Stephen Whitehead, NIAID	Genbank AF038403.1
Dengue virus serotype 2 (strain S-16803)<br>WHO reference strain		Aravinda De Silva, University of North Carolina-Chapel Hill (UNC)	Genbank GU289914
Dengue virus serotype 3 (Sleman/78)		Stephen Whitehead, NIAID	Genbank AY656169

Dengue virus serotype 3 (CH53489)<br>WHO reference strain	Aravinda De Silva, UNC	Genbank DQ863638
Dengue virus serotype 4 Dominica/81	Stephen Whitehead, NIAID	Genbank AY648301
Dengue virus serotype 4 (TVP-376)<br>WHO reference strain	Aravinda De Silva, UNC	Genbank KC963424
Biological Samples		
Human Serum	University of Vermont Vaccine Testing Center and Johns Hopkins Center for Immunization Research	Clinicaltrials.gov identifiers: NCT01072786, NCT02021968
Peripheral blood mononuclear cells (PBMC)	University of Vermont Vaccine Testing Center and Johns Hopkins Center for Immunization Research	Clinicaltrials.gov identifiers: NCT01072786, NCT02021968
Experimental Models: Cell Lines		
African green monkey kidney cells (Vero-81)	Stephen Whitehead, NIAID	RRID:CVC_0059

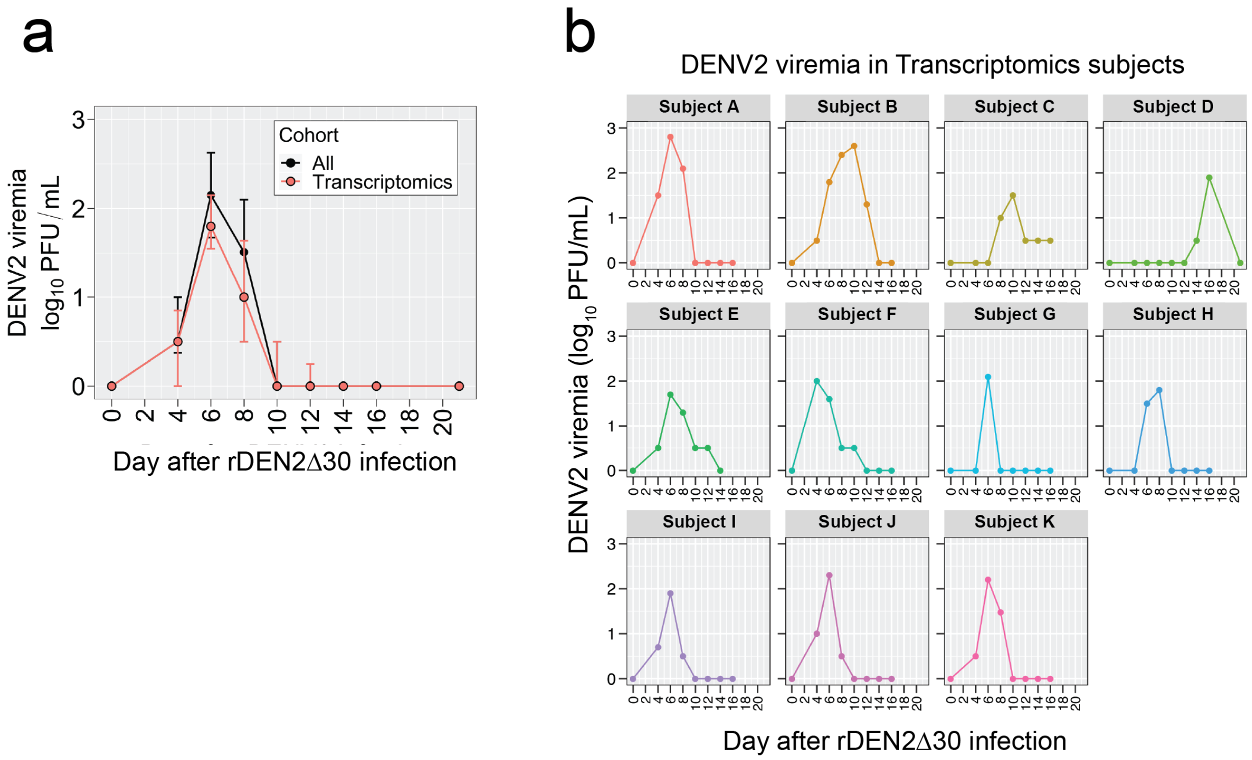

Supplementary Figure 1 | DENV2 viremia after rDEN2Δ30 infection. (a) Comparison of DENV2 serum viremia as assessed by plaque assay (expressed s $\log_{10}$ plaque-forming units/mL of serum) in the parent cohort (All, n = 20) from which a subset was selected for transcriptomics analysis (n = 11). Median ± standard deviation is plotted. (**b**) Individual viremia profiles for each of the subjects in the transcriptomics analysis.

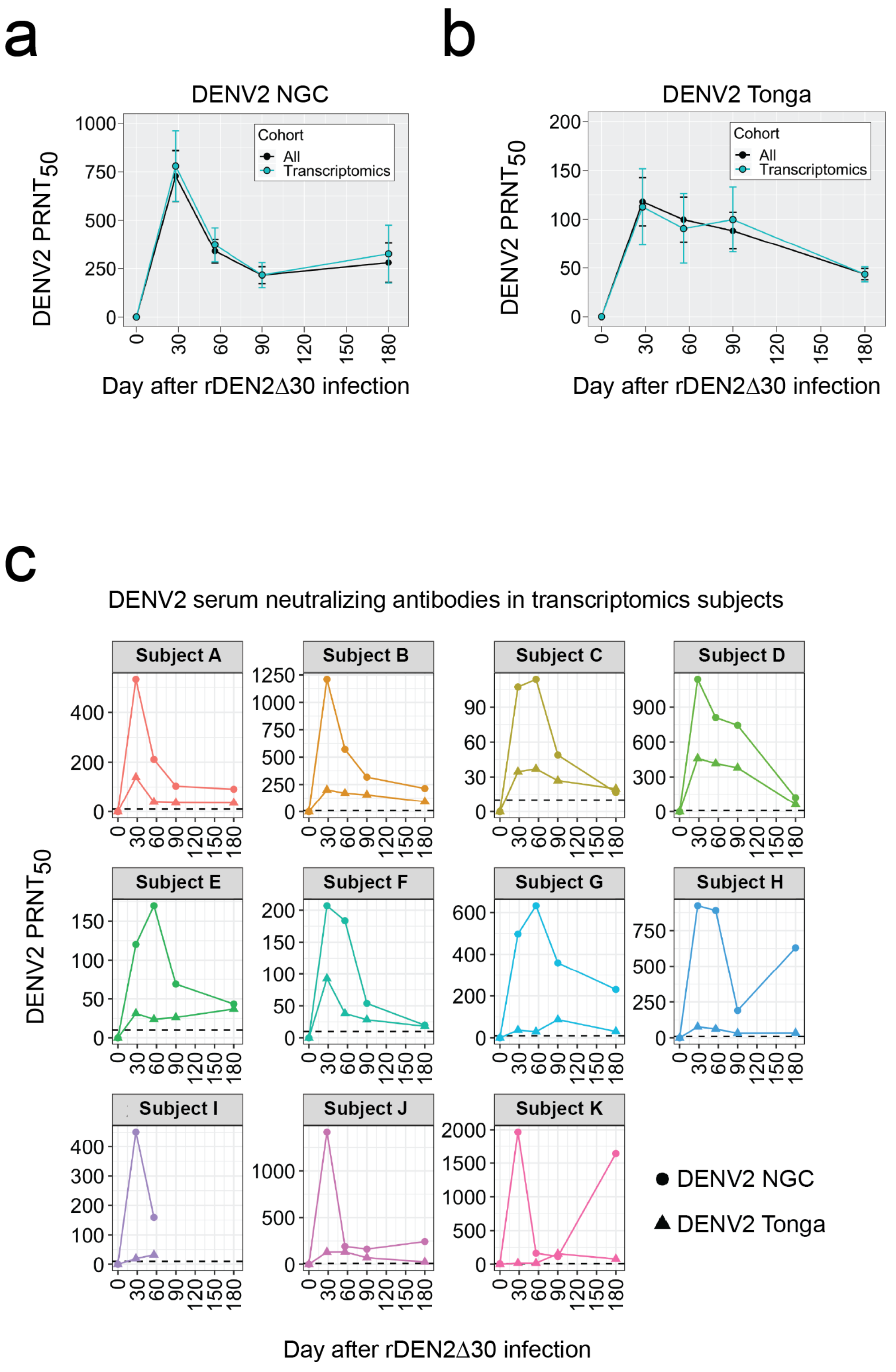

Supplementary Figure 2 | DENV2 serum neutralizing antibodies after rDEN2Δ30 infection. **(a)** Comparison of DENV2 serum neutralizing antibodies as assessed by plaque reduction neutralization titer 50% ($PRNT_{50}$) to (**a**) DEN2 NGC (**b**) DEN2 Tonga in the parent cohort (All,

n = 20) and the subset selected for transcriptomics analysis (n = 11). Mean ± standard deviation values are shown. (**c**) Individual $PRNT_{50}$ profiles for DEN2-NGC (circles) and -Tonga (triangles) for each of the subjects in the transcriptomics analysis.

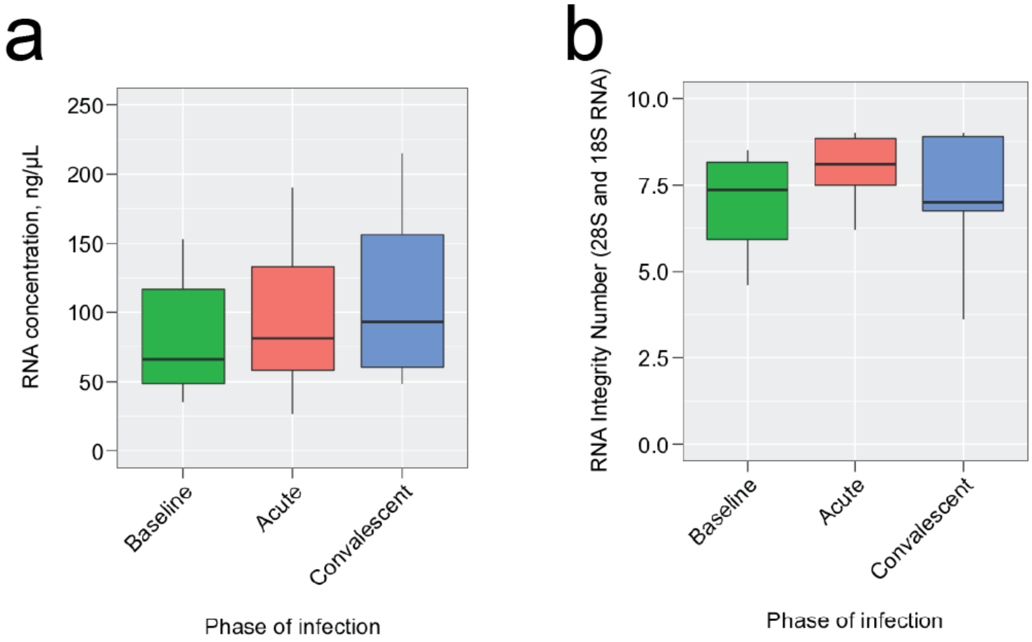

Supplementary Figure 3 | Quantity and quality of whole blood mRNA during rDEN2Δ30 infection. (a) RNA quantity (**a**) and quality (**b**) in samples isolated at Day 0 (baseline), day 8 (acute) and day 28 (convalescent) after primary rDEN2Δ30 infection (see methods). Boxplots show the median (horizontal line), 95% confidence level (box bounds), and minimum and maximum values (whiskers).

Flow cytometric gating strategy and cell population identifiers for Hanley et al.

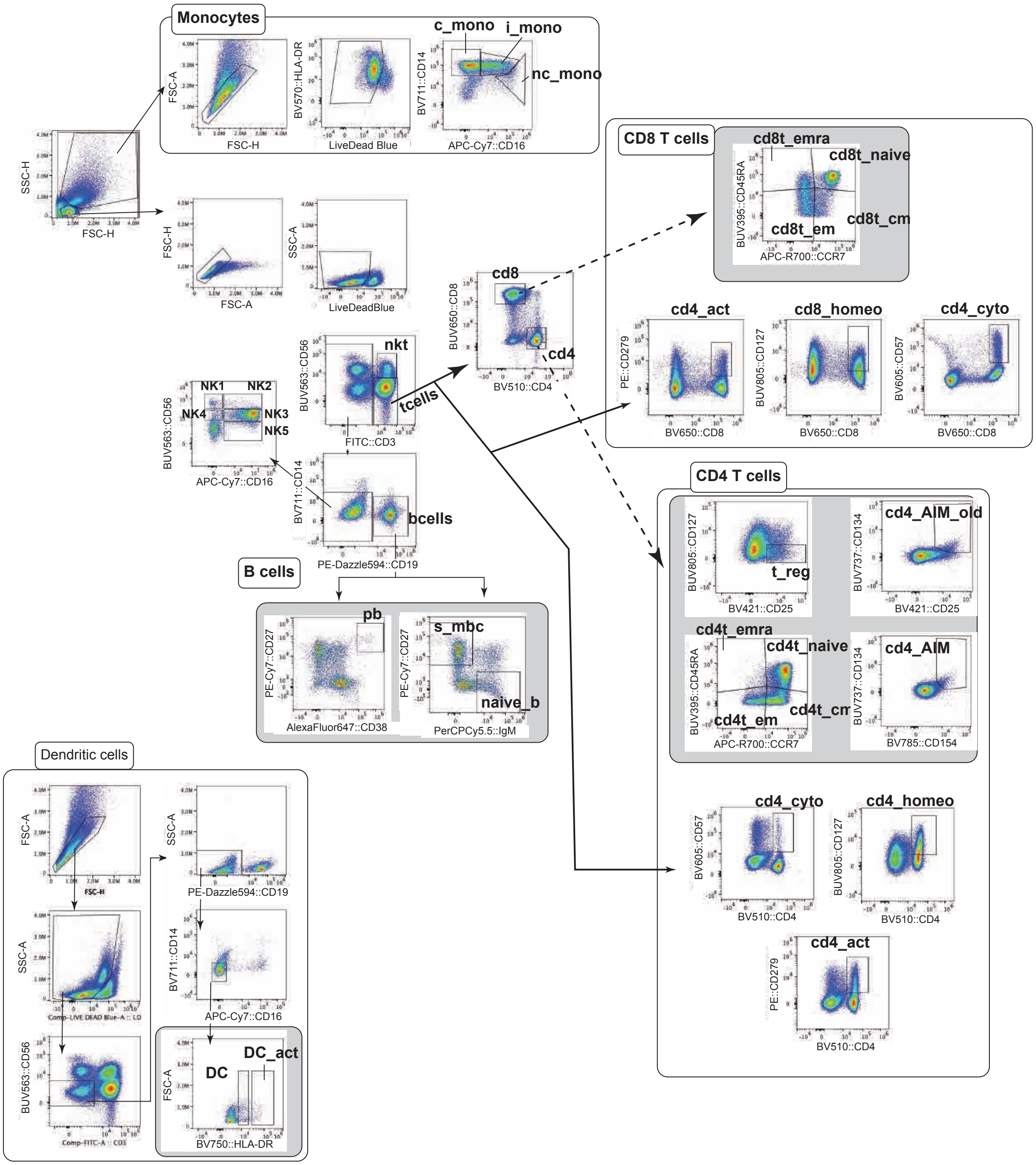

Supplementary Figure 4 | Flow cytometric gating strategy. Labels in bold on plots correspond to graph headings in Figure 4. Lineage-negative (CD3⁻CD19⁻CD56⁻CD14⁻CD16⁻) HLA-DR+ Dendritic cells (**DC**), HLA-DR^{hi} activated DC (**DC_act**); monocytes: classical (**c_mono**), intermediate (**i_mono**), and non-classical (**nc_mono**); natural killer T cells (**nkt**), natural killer cell (**NK)1-5** populations (defined by CD56 and CD16 staining; CD3^{+} T cells (**tcells**), CD4^{+} T

cells (**cd4**), and CD8^{+} T cells (**cd8**); CD8+ T cell populations: Activated CD279^{+} (**cd8_act**), cytotoxic CD57^{+} (**cd8_cyto**), CCR7^{-}CD45RA^{+} T effector re-expressing CD45RA (**cd8t_emra**), CCR7^{+}CD45RA^{+} naïve (**cd8t_naive**), CCR7^{-}CD45RA^{-} effector memory (**cd8t_em**), CCR7^{+}CD45RA^{-} central memory (**cd8t_cm**), and homeostatic CD127^{+} (**cd8_homeo**); CD4^{+} T cell populations: activated CD279^{+} (**cd4_act**), cytotoxic CD57^{+} (**cd4_cyto**), activation-induced marker (AIM)-positive CD154^{+}CD134^{+} (**cd4_AIM**), CD25^{+}CD134^{+} (**cd4_AIM_old**), regulatory T cells CD127^{-}CD25^{+} (**t_regs**), homeostatic CD127^{+} (**cd4_homeo**), CCR7^{-}CD45RA^{+} T effector re-expressing CD45RA (**cd4t_emra**), CCR7^{+}CD45RA^{+} naïve (**cd4t_naive**), CCR7^{-}CD45RA^{-} effector memory (**cd4t_em**), CCR7^{+}CD45RA^{-} central memory (**cd4t_cm**); CD19^{+} B cells (**bcells**), plasmablasts CD19^{+}CD38^{hi}CD27^{hi} (**pb**), IgM^{+}CD27^{-} naïve B cells (**naïve_b**), and IgM^{-}CD27^{+} switched memory B cells (**s_mbc**).

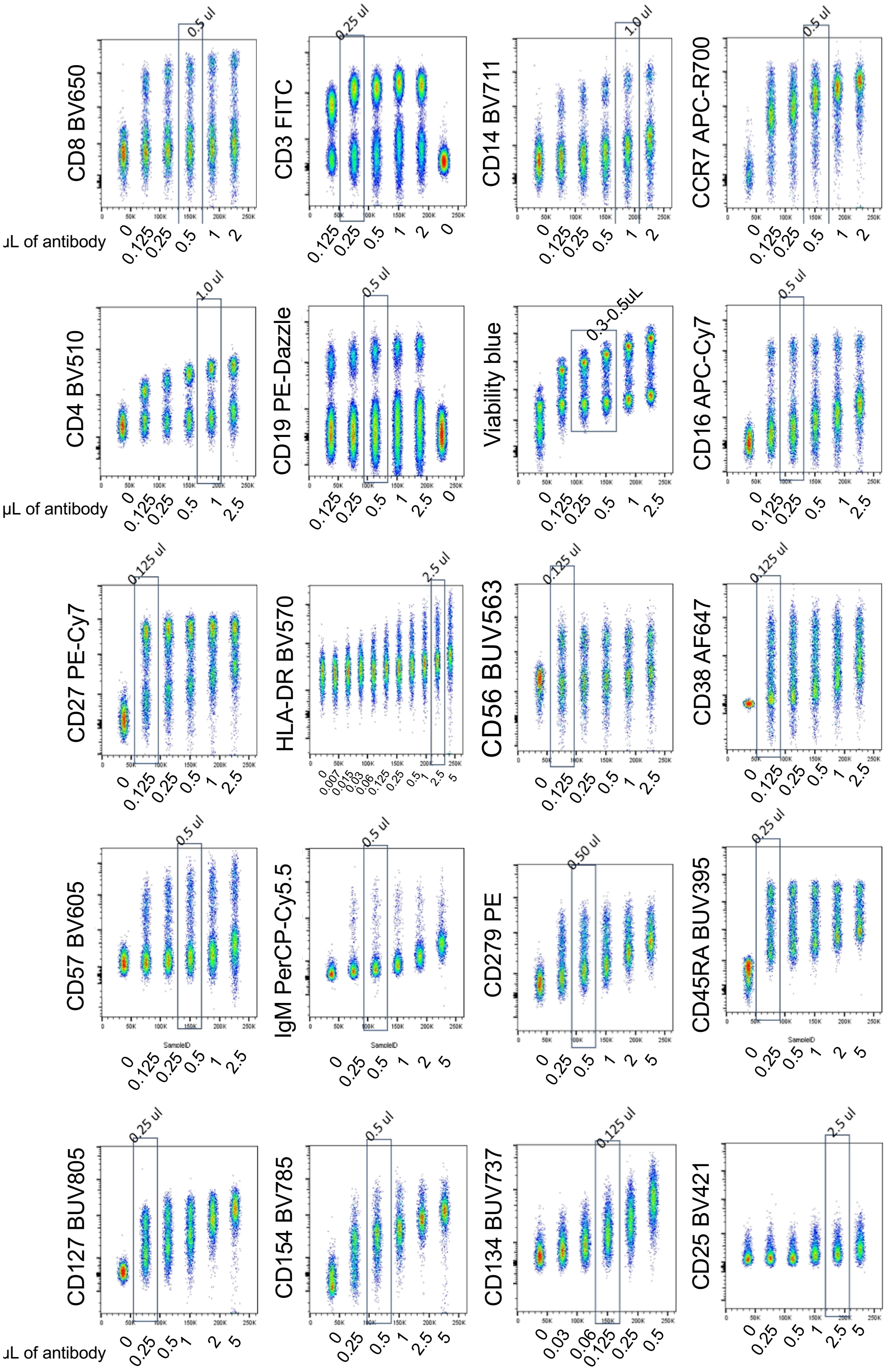

Supplementary Figure 5 | Titration of flow cytometry staining antibodies for multi-color staining panel. A range of dilutions of each single antibody was used to stain 10^6 PBMCs in 50

μL of staining buffer. The fluorescence profiles of each individual antibody dilution (μL of antibody used on x-axis) was plotted on a single plot with fluorescent intensity on y-axis). Based on the brightness and separation of signal (positive or negative or range of staining), the final staining amount for each antibody required in the multi-color panel are indicated by the box and label.
