# Supplementary material for: Immunotranscriptomic profiling the acute and clearance phases of a human challenge dengue virus serotype 2 infection model
Source: Nat Commun. 2021 May 24;12:3054. doi: 10.1038/s41467-021-22930-6 (PMC8144425; doi:10.1038/s41467-021-22930-6)
Supplement: Supplementary file 3 — Reporting Summary [file 41467_2021_22930_MOESM3_ESM.pdf]

nature research

Corresponding author(s): Diehl

Last updated by author(s): February 24, 2021

Reporting Summary

Nature Research wishes to improve the reproducibility of the work that we publish. This form provides structure for consistency and transparency in reporting. For further information on Nature Research policies, see our Editorial Policies and the Editorial Policy Checklist.

Statistics

For all statistical analyses, confirm that the following items are present in the figure legend, table legend, main text, or Methods section.

n/a	Confirmed	
☐	☒	The exact sample size (n) for each experimental group/condition, given as a discrete number and unit of measurement
☐	☒	A statement on whether measurements were taken from distinct samples or whether the same sample was measured repeatedly
☐	☒	The statistical test(s) used AND whether they are one- or two-sided<br>*Only common tests should be described solely by name; describe more complex techniques in the Methods section.*
☐	☒	A description of all covariates tested
☐	☒	A description of any assumptions or corrections, such as tests of normality and adjustment for multiple comparisons
☐	☒	A full description of the statistical parameters including central tendency (e.g. means) or other basic estimates (e.g. regression coefficient) AND variation (e.g. standard deviation) or associated estimates of uncertainty (e.g. confidence intervals)
☐	☒	For null hypothesis testing, the test statistic (e.g. F, t, r) with confidence intervals, effect sizes, degrees of freedom and P value noted<br>*Give P values as exact values whenever suitable.*
☐	☒	For Bayesian analysis, information on the choice of priors and Markov chain Monte Carlo settings
☐	☒	For hierarchical and complex designs, identification of the appropriate level for tests and full reporting of outcomes
☐	☒	Estimates of effect sizes (e.g. Cohen's d, Pearson's r), indicating how they were calculated

Our web collection on statistics for biologists contains articles on many of the points above.

Software and code

Policy information about availability of computer code

Data collection	RNASeq data were collected on an Illumina HiSeq 2000
Data analysis	stringtie (version 1.3.5) was used for genome mapping, BAM files were merged by sample using SAMtools (Version 1.11). Merged BAM files were used for immune deconvolution analysis in CIBERSORT (version 1.06). edgeR (version 3.32.1) was used for differential gene expression, Clustvis (version 2018-12-20) for principal component analysis. KEGG (https://www.genome.jp/kegg/) and DAVID (https://david.ncifcrf.gov) to assign biological functions to the sets of genes that were differentially regulated across different time points. Pathway enrichment analysis was done primarily with DAVID and confirmed with Webgestault (http://www.webgestalt.org) and PantherDB (http://pantherdb.org). All computations and quantifications were performed using the R programming language. Custom scripts can be found at: https://github.com/seandiehl/uvm-vaccine-lab/tree/seandiehl-ncomms_paper or https://doi.org/10.5281/zenodo.4552689

For manuscripts utilizing custom algorithms or software that are central to the research but not yet described in published literature, software must be made available to editors and reviewers. We strongly encourage code deposition in a community repository (e.g. GitHub). See the Nature Research guidelines for submitting code & software for further information.

Data

Policy information about availability of data

All manuscripts must include a data availability statement. This statement should provide the following information, where applicable:

- Accession codes, unique identifiers, or web links for publicly available datasets
- A list of figures that have associated raw data
- A description of any restrictions on data availability

RNASeq data is on the National Center for Biotechnology Information (NCBI) Gene Expression Omnibus under accession number GSE152255 (https://www.ncbi.nlm.nih.gov/geo/query/acc.cgi?acc=GSE152255). All other data are available in the Article file, Supplementary Information or available from the authors

upon reasonable request. Source data are provided with this paper.

Field-specific reporting

Please select the one below that is the best fit for your research. If you are not sure, read the appropriate sections before making your selection.

- [x] Life sciences
- [ ] Behavioural & social sciences
- [ ] Ecological, evolutionary & environmental sciences

For a reference copy of the document with all sections, see nature.com/documents/nr-reporting-summary-flat.pdf

Life sciences study design

All studies must disclose on these points even when the disclosure is negative.

Sample size	We selected a subset of eleven subjects based on viremia onset, duration, and peak titer metrics that matched the variability (quantified as Shannon Entropy) in these metrics of the parent cohort.
Data exclusions	No data were excluded
Replication	There was no difference compared to the parent cohort in terms of demographics (sex, race, and study site) or viral load, onset, or duration viremia characteristics. This is shown in Table S1.
Randomization	Subjects for this study were not randomized by treatment; all were challenged with virus.
Blinding	All subjects were challenged with virus so blinding to intervention was not done.

Reporting for specific materials, systems and methods

We require information from authors about some types of materials, experimental systems and methods used in many studies. Here, indicate whether each material, system or method listed is relevant to your study. If you are not sure if a list item applies to your research, read the appropriate section before selecting a response.

Materials & experimental systems

n/a	Involved in the study	
	x	Antibodies
	x	Eukaryotic cell lines
x		Palaeontology and archaeology
x		Animals and other organisms
	x	Human research participants
	x	Clinical data
x		Dual use research of concern

Methods

n/a	Involved in the study	
x		ChIP-seq
	x	Flow cytometry
x		MRI-based neuroimaging

Antibodies

Antibodies used	All antibodies used are described with clone, conjugate, amount used, manufacturer, catalog number, Lot#. This information is also in the Supplemental Table S2. These are: anti-human CD3 (UCHT1, FITC-conjugated, 0.25 µL/test, Biolegend, Cat. #300406, Lot #B279208); anti-human CD4 (OKT4, BV510-conjugated, 1 µL/test, Biolegend, Cat. #317444, Lot #B248141); anti-human CD8 (RPA-T8, BV650-conjugated, 0.5 µL/test, Biolegend, Cat. #301041, Lot #B275821); anti-human CD14 (M5E2, BV711-conjugated, 1 µL/test, Biolegend, Cat. #301837, Lot #B275829); anti-human CD16 (3G8, APC-Cy7-conjugated, 0.25 µL/test, Biolegend, Cat. #302017, Lot #B295391); anti-human CD19 (HIB19, PE-Dazzle594-conjugated, 0.5 µL/test, Biolegend, Cat. #302252, Lot #B277039); anti-human CD25 (M-A251, BV421-conjugated, 0.5 µL/test, Biolegend, Cat. #356113, Lot #B301467); anti-human CD27 (M-T271, PE-Cy7-conjugated, 0.125 µL/test, Biolegend, Cat. #356412, Lot #B279971); anti-human CD38 (HIT2, Alexa Fluor 647-conjugated, 0.125 µL/test, Biolegend, Cat. #303514, Lot #B233813); anti-human CD45RA (HI100, BUV395-conjugated, 0.25 µL/test, BD OptiBuild, Cat. #740298, Lot #0293615); anti-human CD56 (NCAM16.2, BUV563-conjugated, 0.125 µL/test, BD Horizon, Cat. #612929, Lot #0044064); anti-human CD57 (QA17A04, BV605-conjugated, 0.5 µL/test, Biolegend, Cat. #393303, Lot #270939); anti-human CD127 (HIL-7R-M21, BUV805-conjugated, 0.25 µL/test, BD OptiBuild, Cat. #748486, Lot #0294340); anti-human CD134 (ACT35, BUV737-conjugated, 0.125 µL/test, BD OptiBuild, Cat. #749286, Lot #0294339); anti-human CD154 (24-31, BV785-conjugated, 0.5 µL/test, Biolegend, Cat. #310841, Lot #B264809); anti-human HLA-DR (L243, BV570-conjugated, 2.5 µL/test, Biolegend, Cat. #307637, Lot #B314475); anti-human IgM (MHM-88, PerCP-Cy5.5-conjugated, 0.5 µL/test, Biolegend, Cat. #314512, Lot #B231968); anti-human CCR7 (2-L1-A, APC-R700-conjugated, 0.5 µL/test, BD Horizon, Cat. #566767, Lot #0283646); anti-human CD279 (EH12.2H7, PE-conjugated, 0.5 µL/test, Biolegend, Cat. #329905, Lot #B252642).
Validation	• Anti-human CD3 (UCHT1, FITC-conjugated, Biolegend, Cat. #300406, https://www.biolegend.com/en-us/products/fitc-anti-human-cd3-antibody-863, RRID: AB_314060<br>• Anti-human CD4 (OKT4, BV510-conjugated, Biolegend, Cat. #317444, https://www.biolegend.com/en-us/products/brilliant-

violet-510-anti-human-cd4-antibody-8010, RRID: AB_2561866
• Anti-human CD8 (RPA-T8, BV650-conjugated, Biolegend, Cat. #301041, https://www.biolegend.com/en-us/products/brilliant-violet-650-anti-human-cd8a-antibody-7652, RRID: AB_11125174
• Anti-human CD14 (M5E2, BV711-conjugated, Biolegend, Cat. #301837, https://www.biolegend.com/en-us/products/brilliant-violet-711-anti-human-cd14-antibody-7932, RRID: AB_11218986
• Anti-human CD16 (3G8, APC-Cy7-conjugated, Biolegend, Cat. #302017, https://www.biolegend.com/en-us/products/apc-cyanine7-anti-human-cd16-antibody-1904, RRID: AB_314217
• Anti-human CD19 (HIB19, PE-Dazzle594-conjugated, Biolegend, Cat. #302252, https://www.biolegend.com/en-us/products/pe-dazzle-594-anti-human-cd19-antibody-9783, RRID: AB_2563560
• Anti-human CD25 (M-A251, BV421-conjugated, Biolegend, Cat. #356113, https://www.biolegend.com/en-us/products/brilliant-violet-421-anti-human-cd25-antibody-8589, RRID: AB_2562163
• Anti-human CD27 (M-T271, PE-Cy7-conjugated, Biolegend, Cat. #356412, https://www.biolegend.com/en-us/products/pe-cyanine7-anti-human-cd27-antibody-8640, RRID: AB_2562258
• Anti-human CD38 (HIT2, Alexa Fluor 647-conjugated, Biolegend, Cat. #303514, https://www.biolegend.com/en-us/products/alexa-fluor-647-anti-human-cd38-antibody-3264, RRID: AB_493090
• Anti-human CD45RA (HI100, BUV395-conjugated, BD OptiBuild, Cat. #740298, https://www.bdbiosciences.com/us/reagents/research/antibodies-buffers/immunology-reagents/anti-human-antibodies/cell-surface-antigens/buv395-mouse-anti-human-cd45ra-hi100/p/740298, RRID: AB_2740037
• Anti-human CD56 (NCAM16.2, BUV563-conjugated, BD Horizon, Cat. #612929, https://www.bdbiosciences.com/us/reagents/research/antibodies-buffers/immunology-reagents/anti-human-antibodies/cell-surface-antigens/buv563-mouse-anti-human-cd56-ncam162-also-known-as-ncam-16/p/612929, RRID: AB_2870213
• Anti-human CD57 (QA17A04, BV605-conjugated, Biolegend, Cat. #393303, https://www.biolegend.com/en-us/products/brilliant-violet-605-anti-human-cd57-recombinant-antibody-15480, RRID: AB_2728425
• Anti-human CD127 (HIL-7R-M21, BUV805-conjugated, BD OptiBuild, Cat. #748486, https://www.bdbiosciences.com/us/reagents/research/antibodies-buffers/immunology-reagents/anti-human-antibodies/cell-surface-antigens/buv805-mouse-anti-human-cd127-hil-7r-m21/p/748486, RRID: AB_2872901
• Anti-human CD134 (ACT35, BUV737-conjugated, BD OptiBuild, Cat. #749286, https://www.bdbiosciences.com/us/reagents/research/antibodies-buffers/immunology-reagents/anti-human-antibodies/cell-surface-antigens/buv737-mouse-anti-human-cd134-act35-also-known-as-ber-act35/p/749286, RRID: AB_2873661
• Anti-human CD154 (24-31, BV785-conjugated, Biolegend, Cat. #310841, https://www.biolegend.com/en-us/products/brilliant-violet-785-anti-human-cd154-antibody-12994, RRID:AB_2572186
• Anti-human HLA-DR (L243, BV570-conjugated, Biolegend, Cat. #307637, https://www.biolegend.com/en-us/products/brilliant-violet-570-anti-human-hla-dr-antibody-7457, RRID: AB_10895753
• Anti-human IgM (MHM-88, PerCP-Cy5.5-conjugated, Biolegend, Cat. #314512, https://www.biolegend.com/en-us/products/percp-cyanine5-5-anti-human-igm-antibody-4520, RRID: AB_2076098
• Anti-human CCR7 (2-L1-A, APC-R700-conjugated, BD Horizon, Cat. #566767, https://www.bdbiosciences.com/us/reagents/research/antibodies-buffers/immunology-reagents/anti-human-antibodies/cell-surface-antigens/apc-r700-mouse-anti-human-ccr7-cd197-2-l1-a/p/566767, RRID:AB_2869856
• Anti-human CD279 (EH12.2H7, PE-conjugated, Biolegend, Cat. #329905, https://www.biolegend.com/en-us/products/pe-anti-human-cd279-pd-1-antibody-4412, AB_940481

Eukaryotic cell lines

Policy information about cell lines

Cell line source(s)	African green monkey kidney cells (Vero-81) were obtained from Stephen Whitehead, NIAID. Available at ATCC (https://www.atcc.org/products/all/ccl-81.aspx), RRID:CVC_0059
Authentication	Cell lines were authenticated by their ability to produce and be infected by dengue viruses. Cells were not further authenticated.
Mycoplasma contamination	Vero cells were tested for mycoplasma contamination by PCR amplification in cell culture supernatants and were negative.
Commonly misidentified lines (See ICLAC register)	No commonly misidentified cells lines were used in this research

Human research participants

Policy information about studies involving human research participants

Population characteristics	Healthy flavivirus-naive adult men and women aged 18-50 were enrolled.
Recruitment	Recruitment was by local advertisement and word-of-mouth. Subjects were selected on the basis of meeting inclusion criteria, completion of informed consent, and ability to adhere to study protocol. Since this was a randomized blinded trial with laboratory-defined outcomes, self selection bias for initial participation was not a concern.
Ethics oversight	Institutional Review Boards at Johns Hopkins University and the University of Vermont provided oversight and approval

Note that full information on the approval of the study protocol must also be provided in the manuscript.

Clinical data

Policy information about clinical studies

All manuscripts should comply with the ICMJE guidelines for publication of clinical research and a completed CONSORT checklist must be included with all submissions.

Clinical trial registration	ClinicalTrials.gov NCT02021968
Study protocol	The full study protocol was originally published as Kirkpatrick et al. 2016. Sci. Trans. Med. 8(330): 330ra36
Data collection	Clinical data were collected at Johns Hopkins University, Baltimore, MD and at the Larner College of Medicine at University of Vermont, Burlington, VT in November 2013 through February 2014. Recruitment occurred on an ongoing basis, opening in April 2013.
Outcomes	Primary outcome was development of serum DENV2 viremia and secondary outcomes included clinical symptoms including rash, headache, myalgia, arthralgia, leukopenias.

Flow Cytometry

Plots

Confirm that:

- [x] The axis labels state the marker and fluorochrome used (e.g. CD4-FITC).
- [x] The axis scales are clearly visible. Include numbers along axes only for bottom left plot of group (a 'group' is an analysis of identical markers).
- [x] All plots are contour plots with outliers or pseudocolor plots.
- [x] A numerical value for number of cells or percentage (with statistics) is provided.

Methodology

Sample preparation	Cryopreserved PBMC were thawed in IMDM (8% FBS) and rested overnight at 37°C. On day of sample staining, cells were washed in PBS, counted and stained with viability dye (Invitrogen) before treated with Fc blocker and monocyte blocker (Biolegend) according to manufacturers' indication. $1x10^6$ cells were resuspended in 50µL FACS buffer (1% FBS in PBS) and added to antibody cocktail prepared in Brilliant stain buffer (BD Horizon). Samples were stained in the dark for 30 minutes at 4°C, then washed twice with FACS buffer. All samples were acquired on Cytek Aurora. The antibodies and reagents used in this study are listed in the Supplement.
Instrument	Cytek Aurora
Software	Flow cytometry data was collected using Cytek SpectroFlo® software (version 2.2.0) and outputted as unmixed FCS 3.1 files, and subsequently analyzed with FlowJo v10 to determine population frequencies.
Cell population abundance	Cell populations are defined by the gating strategy schematic in the Supplemental Figure S4 and are expressed as percent of relevant parent population as indicated in the Methods.
Gating strategy	Gating was performed based on non-binding control antibodies and fluorescence minus one (FMO) controls in PBMCs. Dead cells and cell debris were excluded by scatter gates, size, and viability staining.

- [x] Tick this box to confirm that a figure exemplifying the gating strategy is provided in the Supplementary Information.
